# Analysis of factors affecting long-term quality of life in children on home mechanical ventilation: a 4-year prospective observational cohort study

**DOI:** 10.1186/s13052-025-01920-0

**Published:** 2025-03-13

**Authors:** Yingying Zhang, Yan Qin, Pan Liu, Yuxin Liu, Zhengzheng Zhang, Yun Jin, Guoping Lu, Jing Hu, Weiming Chen

**Affiliations:** 1 Department of Critical Care Medicine, Children’s Hospital of Fudan University, No.399 Wanyuan Road, Shanghai, 201102 China; 2 Department of Nursing, Children’s Hospital of Fudan University, No.399 Wanyuan Road, Shanghai, 201102 China

**Keywords:** Quality of life, Home mechanical ventilation, Prolonged mechanical ventilation, Influencing factors, Prospective study

## Abstract

**Background:**

Home mechanical ventilation improves survival of critically ill children but partially affects quality of life. Studies in China have more often analyzed the risk factors for death from prolonged mechanical ventilation in hospitalized children while less attention has been paid to children with home mechanical ventilation. This study aimed to describe the quality of life of children with home mechanical ventilation and the influencing factors.

**Methods:**

It was a prospective cohort study. The cohort population was children undergoing prolonged mechanical ventilation in the Pediatric Intensive Care Unit, with the outcome of whether they were alive or dead at 1-year follow-up after discharge. Standardized scores for quality of life were calculated using TNO-AZL Children’s Health-Related Quality of Life and TNO-AZL Preschool Children Quality of Life. Multiple linear regression was used to analyze the factors affecting the quality of life.

**Results:**

A total of 106 children were included, and 11 children (10.38%) died within one year after discharge. The mean age was 8.26 ± 4.10 years, and the hospitalization days was 68.46 ± 34.23. Child self-care had a significant effect on the risk of death one year after discharge, with higher Barthel self-care scores associated with a lower risk of death. There was a statistically significant difference in quality-of-life scores between the non-home and home mechanical ventilation groups, whereas tracheotomy or not had no effect. 81.57% of the surviving children with home mechanical ventilation were placed on invasive mechanical ventilation, with a mean ventilation duration of 19.94 h/d and a pressure-controlled mode primarily. Home invasive mechanical ventilation, age, and Barthel self-care score were independent influences on children’s quality of life scores.

**Conclusion:**

The long-term survival rate of children who transitioned to home mechanical ventilation in Shanghai, China, was higher than the international average. Most children were discharged to home invasive mechanical ventilation, decreasing their quality of life. It needs to continue standardizing the post-discharge management procedures and explore how to better transition to home non-invasive mechanical ventilation. It’s necessary to describe the parents’ quality of life with home mechanical ventilated children and its impact on child outcomes.

## Background

With the development of pediatric critical care medicine and therapeutic techniques, prolonged mechanical ventilation has increased the survival rate of critically ill children, especially with the shift from prolonged mechanical ventilation in hospitals to home mechanical ventilation (HMV). It reported that the estimated prevalence of pediatric HMV was calculated at 7.4/100 000 children [1]. Either way, it has significant social, psychological, physical, and financial consequences for the child and their family. Health-related quality of life (HRQoL) questionnaires are multidimensional tools that explore aspects of patients’ lives and assess physical, mental, and social well-being, which are not usually covered by other diagnostic tools. Numerous studies have objectively evaluated the quality of life of HMV children, and perceived QoL by children with HMV and their families is lower than that of healthy children [2–4]. Studies in China have focused more on the clinical distribution characteristics [5], risk factors for death of prolonged mechanical ventilation children [6], and the best mode to shift to home mechanical ventilation [7]. Therefore, this study aims to analyze the survival rate of children on prolonged mechanical ventilation one year after discharge and the factors influencing their quality of life. It also explores effective follow-up management strategies to help improve the quality of life of these children and promote the rational allocation of social resources.

## Methods

### Study design and setting

It was a prospective cohort study. A cohort of children undergoing prolonged mechanical ventilation in the Pediatric Intensive Care Unit (PICU) of Children’s Hospital of Fudan University (CHFU) was used to describe their quality of life (QoL) and explore the affected factors, with the outcome of whether they were alive or dead at the 1-year post-discharge follow-up.

Prolonged mechanical ventilation (PMV) is continuous invasive or noninvasive for ≥ 21 days and ≥ 6 h of daily mechanical ventilation. Home mechanical ventilation (HMV) is a noninvasive or invasive mechanical ventilation via tracheotomy performed by a patient at home or in a care facility outside the hospital for ≥ 3 months.

### Inclusion and exclusion criteria

Inclusion criteria were (i) children with PMV admitted to the PICU from January 1, 2020, to August 31, 2023; (ii) > 28 days and ≤ 18 years; (iii) accessible follow-up outcomes one year after discharge; and (iv) informed consent from the child’s caregivers. The exclusion criteria were in-hospital brain death cases following “the Criteria and Practice for the Determination of Childhood Brain Death in China.”

### Outcomes

The primary outcome indicator was the QoL score at one year of discharge. Children aged 6–15 years were scored using the TNO-AZL Children’s Health-Related Quality of Life (TACQOL) [8]. This questionnaire was designed by Vogels T et al. and can be used to assess health-related quality of life in children with various chronic diseases. The questionnaire contained a total of 56 entries in seven dimensions: physical condition, motor function, self-care ability, cognitive ability, interaction ability, positive emotion, and negative emotion, and was scored on a 4-point Likert scale, with the entries summed up to obtain a total score, with higher scores indicating a better quality of life. For children aged 2 months-5 years, a derivative version of TACQOL, the TNO-AZL Preschool Children Quality of Life (TAPQOL) [9], was used. The scale, designed by Fekkes, was a multidimensional questionnaire measuring parental assessment of children’s health-related quality of life. It measured functional problems by weighting infants’ and children’s handling of emotional issues on each dimension and contains 43 entries on 12 dimensions, including sleep, appetite, lung function, stomach function, skin function, motor function, social function, problematic behaviors, communication function, anxiety, positive emotions, and vitality. A Likert 4-point scale was used, and the entries were summed up to obtain a total score, with higher scores indicating a better quality of life.

### Data collection

(i) Basic information about the child at admission to the PICU, including age, gender, and type of primary disease; (ii) Information about the child’s treatment in the PICU, including whether vasoactive drugs were used, the total length of mechanical ventilation during hospitalization, and the number of tracheal intubations; (iii) Information about the child’s discharge from the hospital, including the number of days and costs of PICU hospitalization, whether or not the child’s caregivers received mechanical ventilation training and assessment before discharge, whether or not the child was mechanically ventilated or tracheotomized at home after discharge; (iv) Information about the child’s post-discharge care, including the primary caregiver after discharge and their education level, the number of children in the family, whether an infection occurred within 90 days after discharge, whether the child was readmitted to the hospital within 90 days after discharge, the monthly post-discharge care costs, the family’s average annual income, the swallowing ratings, the feeding practices, the Barthel Self-Care Rating scores, and the QoL scores.

### Statistical analysis

SPSS 27.0 was used for statistical analysis. If the sample size was greater than 50, the K-S one-sample test was used to test the normality of continuous variables; otherwise, the S-W test was used. Normally distributed continuous variables were expressed as M ± SD, and *t*-tests were used for group comparisons. Non-normally distributed data were expressed as M (P25, P75), and the Mann-Whitney *U* test was used to compare groups. Categorical variables were expressed as n (%). Comparisons between groups were performed using the χ2 test, and the Fisher exact probability test was used if the individual expected frequency of the incidence rate or the component ratio was < 5. Differences were considered to be statistically significant at *P* < 0.05. For the comparison of QoL scores, because the TACQOL scale was used for children aged 6–15 years and the TAPQOL scale was used for children aged 2 months-5 years, and the total number of entries of the two scales was inconsistent, the QoL scores of the children in the two age groups were firstly tested for normal distribution, and if they conformed to the normal distribution, the standardized scores were calculated (standardized scores = (raw scores - mean)/standard deviation), and then the difference was tested. Multiple linear regression was used to analyze the influencing factors of the QoL scores, and the difference was considered statistically significant at *P* < 0.05.

## Results

### Analysis of basic information

A total of 106 cases of children with PMV were included, 95 cases in the survival group, and 11 cases (10.38%) died within one year. The average age of children with PMV was 8.26 ± 4.10 years, the average hospitalization days were 68.46 ± 34.23 days, and the average length of mechanical ventilation during the hospitalization period was 939.11 ± 565.15 h. The types of primary diseases were mainly central respiratory failure caused by various reasons, such as brain tumor, epilepsy, mitochondrial encephalopathy, craniocerebral injury, spinal cord injury, etc., followed by neuromuscular disorders such as congenital muscular dystrophy, spinal muscular atrophy, and Guillain-Barre syndrome, and then by lower respiratory tract diseases. The Barthel Self-Care Rating scores of the children in the survival group were significantly higher than those in the death group. There were differences between the survival and death groups in terms of post-discharge primary caregiver, monthly care costs, and whether or not the child was readmitted to the hospital within 90 days of discharge, while the differences in the remaining indicators were not statistically significant, as shown in Table 1.

**Table 1:** Summary of basic information

Variables	Total (*n* = 106)	Survival (*n* = 95)	Dead (*n* = 11)	Statistical value	*P*
**Age (years)**	8.00 (5.00, 11.00)	8.00 (5.00, 11.00)	7.00 (5.50, 11.00)	Z=-0.09	0.925
**Total length of hospitalization mechanical ventilation (hours)**	768.00 (552.75, 1224.00)	744.00 (540.00, 1176.00)	1056.00 (708.00, 1404.00)	Z=-1.53	0.125
**Costs of PICU hospitalization (RMB)**	247500.00 (169250.00, 383184.00)	245000.00 (168500.00, 389489.00)	258000.00 (177800.00, 346000.00)	Z=-0.10	0.922
**Barthel Self-Care Rating scores**	45.00 (30.00, 83.75)	55.00 (30.00, 87.50)	20.00 (20.00, 30.00)	Z=-3.76	**< 0.001**
**Days of PICU hospitalization**	59.50 (43.25, 85.50)	61.00 (42.50, 86.50)	54.00 (44.50, 65.50)	Z=-0.74	0.462
**Gender**				χ²=3.03	0.082
Male	66 (62.26)	56 (58.95)	10 (90.91)		
Female	40 (37.74)	39 (41.05)	1 (9.09)		
**Vasoactive drugs**				χ²=1.16	0.281
Used	77 (72.64)	67 (70.53)	10 (90.91)		
Non-used	29 (27.36)	28 (29.47)	1 (9.09)		
**Primary disease**				-	0.894
Neuromuscular disorders	17 (16.04)	16 (16.84)	1 (9.09)		
Central respiratory failure	66 (62.26)	57 (60.00)	9 (81.82)		
Upper airway disease	4 (3.77)	4 (4.21)	0 (0.00)		
Lower respiratory tract diseases	14 (13.21)	13 (13.68)	1 (9.09)		
Others	5 (4.72)	5 (5.26)	0 (0.00)		
**Number of tracheal intubations**				-	0.196
1	52 (49.06)	43 (45.26)	9 (81.82)		
2	44 (41.51)	42 (44.21)	2 (18.18)		
3	9 (8.49)	9 (9.47)	0 (0.00)		
4	1 (0.94)	1 (1.05)	0 (0.00)		
**Primary caregiver after discharge**				-	**0.004**
Father	33 (31.13)	27 (28.42)	6 (54.55)		
Mother	61(57.55)	59 (62.11)	2 (18.18)		
Grandparents	9 (8.49)	8 (8.42)	1 (9.09)		
Specialist staff	3 (2.83)	1 (1.05)	2 (18.18)		
**Number of children in the family**				χ²=0.19	0.659
One child	69 (65.09)	63 (66.32)	6 (54.55)		
2 or more children	37 (34.91)	32 (33.68)	5 (45.45)		
**Primary caregiver’s education level**				χ²=0.69	0.406
Below undergraduate	70 (66.04)	61 (64.21)	9 (81.82)		
Undergraduate and above	36 (33.96)	34 (35.79)	2 (18.18)		
**Whether or not the child’s caregivers received mechanical ventilation training before discharge**				χ²=1.13	0.287
Yes	77 (72.64)	71 (74.74)	6 (54.55)		
No	29 (27.36)	24 (25.26)	5 (45.45)		
**Whether or not the child’s caregivers received mechanical ventilation assessment before discharge**				χ²=0.66	0.415
Yes	65 (61.32)	60 (63.16)	5 (45.45)		
No	41 (38.68)	35 (36.84)	6 (54.55)		
**HMV**				χ²=0.27	0.605
Yes	50 (47.17)	44 (46.32)	6 (54.55)		
No	56 (52.83)	51 (53.68)	5 (45.45)		
**Tracheotomy**				χ²=0.00	1.000
Yes	58 (54.72)	52 (54.74)	6 (54.55)		
No	48 (45.28)	43 (45.26)	5 (45.45)		
**Whether an infection occurred within 90 days after discharge**				χ²=3.07	0.080
Yes	46 (43.40)	38 (40.00)	8 (72.73)		
No	60 (56.60)	57 (60.00)	3 (27.27)		
**Whether the child was readmitted to the hospital within 90 days after discharge**				χ²=11.21	**< 0.001**
Yes	42 (39.62)	32 (33.68)	10 (90.91)		
No	64 (60.38)	63 (66.32)	1 (9.09)		
**Monthly care costs after discharge**				-	**0.031**
< 3000 RMB	44 (41.51)	43 (45.26)	1 (9.09)		
3000–5000 RMB	35 (33.02)	28 (29.47)	7 (63.64)		
> 5000 RMB	27 (25.47)	24 (25.26)	3 (27.27)		
**Family’s average annual income**				-	0.220
< 10,000 RMB	29 (27.36)	24 (25.26)	5 (45.45)		
10,000–20,000 RMB	37 (34.91)	33 (34.74)	4 (36.36)		
> 20,000 RMB	40 (37.74)	38 (40.00)	2 (18.18)		
**Swallowing ratings**				-	0.074
Level 1	29 (27.36)	25 (26.32)	4 (36.36)		
Level 2	11 (10.38)	11 (11.58)	0 (0.00)		
Level 3	13 (12.26)	13 (13.68)	0 (0.00)		
Level 4	1 (0.94)	3 (3.16)	0 (0.00)		
Level 5	3 (2.83)	7 (7.37)	3 (27.27)		
Level 6	10 (9.43)	10 (10.53)	1 (9.09)		
Level 7	11 (10.38)	26 (27.37)	2 (18.18)		
Level 8	28 (26.42)	0 (0.00)	1 (9.09)		
**Feeding practices**				-	0.151
Normal feeding	37 (34.91)	36 (37.89)	1 (9.09)		
Nasogastric/enteric tube feeding	45 (42.45)	38 (40.00)	7 (63.64)		
Normal feeding mixed nasogastric/enteric tube feeding	11 (10.38)	10 (10.53)	1 (9.09)		
Normal feeding mixed gastrostomy feeding	13 (12.26)	11 (11.58)	2 (18.18)		

Z: Mann-Whitney test, χ²: Chi-square test, -: Fisher exact

### QoL scores at one year of discharge for surviving children in different subgroups

Ninety-five children with PMV who survived for one year after discharge were grouped according to whether they were tracheotomized or not and whether they underwent HMV or not. Among them, four tracheotomized children and six HMV children were excluded due to data inaccessibility, and the remaining were divided into tracheotomy (48 cases, 52.75%) and non-tracheotomy groups (43 cases, 47.25%), HMV (38 cases, 42.70%) and non-HMV groups (51 cases, 57.30%). The QoL scores of the children in both groups were shown in Table 2. There was no statistical difference in the QoL scores between the tracheotomy and non-tracheotomy groups. In contrast, the QoL scores in the non-HMV group were significantly higher than those in the HMV group.

**Table 2:** Differences in QoL scores between groups

Variables	Tracheotomy (*n* = 48)	Non-tracheotomy (*n* = 43)	Statistical value	*P*	HMV (*n* = 38)	Non-HMV (*n* = 51)	Statistical value	*P*
QoL scores	-0.33 (-0.79, 0.71)	-0.08 (-0.63, 1.04)	Z=-1.28	0.202	-0.30 ± 1.08	0.22 ± 0.89	t=-2.50	**0.014**

### Factors influencing QoL scores in children with HMV

Of the 38 surviving children with HMV, 31 had invasive mechanical ventilation, and 7 had noninvasive mechanical ventilation. The average duration of invasive mechanical ventilation was 19.94 h/d, and the primary mode was pressure-controlled; the average duration of noninvasive mechanical ventilation was 12.29 h/d, and the main mode was respiratory/time-controlled.

The linear relationship between multiple independent variables and the dependent variable was explored using the QoL score as the dependent variable. Eleven independent variables were finally included for multiple linear regression analysis. As the results are shown in Table 3, home invasive mechanical ventilation, age, and Barthel Self-Care Rating scores were independent influences on children’s quality of life scores. Invasive mechanical ventilation led to a decrease in QoL, and the older the age and the higher the Barthel Self-Care Rating score, the better the QoL of the children, and the linear relationship of the regression equation was significant (F = 12.498, *P* < 0.001).

**Table 3:** Factors influencing QoL scores in children with HMV

Variables	Single factor						Multi-factor				
	β	S. E	t	*P*	β (95%CI)		β	S. E	t	*P*	β (95%CI)
**Whether the primary caregiver was trained in mechanical ventilation care before discharge**											
Yes					0.00 (Reference)						0.00 (Reference)
No	0.51	0.24	2.13	**0.036**	0.51 (0.04 ~ 0.99)		0.03	0.20	0.17	0.869	0.03 (-0.35 ~ 0.42)
**Type of home mechanical ventilation**											
No mechanical ventilation					0.00 (Reference)						0.00 (Reference)
Invasive mechanical ventilation	-0.76	0.21	-3.64	**< 0.001**	-0.76 (-1.17 ~ -0.35)		-0.36	0.17	-2.10	**0.039**	-0.36 (-0.70 ~ -0.02)
Non-invasive mechanical ventilation	0.55	0.37	1.48	0.142	0.55 (-0.18 ~ 1.27)		0.02	0.29	0.06	0.953	0.02 (-0.55 ~ 0.58)
**Primary caregiver after discharge**											
Parents					0.00 (Reference)						
Grandparents	0.07	0.36	0.21	0.836	0.07 (-0.63 ~ 0.77)						
Specialist staff	-0.18	0.38	-0.49	0.626	-0.18 (-0.92 ~ 0.55)						
**Primary caregiver’s education level**											
Below undergraduate					0.00 (Reference)						
Undergraduate and above	-0.30	0.22	-1.39	0.169	-0.30 (-0.73 ~ 0.13)						
**Whether an infection occurred within 90 days after discharge**											
Yes					0.00 (Reference)						0.00 (Reference)
No	0.54	0.21	2.54	**0.013**	0.54 (0.12 ~ 0.96)		-0.33	0.19	-1.74	0.086	-0.33 (-0.71 ~ 0.04)
**Monthly care costs after discharge**											
< 3000 RMB					0.00 (Reference)						
3000–5000 RMB	-0.29	0.24	-1.18	0.242	-0.29 (-0.77 ~ 0.19)						
> 5000RMB	-0.47	0.27	-1.76	0.081	-0.47 (-0.99 ~ 0.05)						
**Family’s average annual income**											
< 10,000 RMB					0.00 (Reference)						
10,000–20,000 RMB	-0.49	0.27	-1.83	0.071	-0.49 (-1.01 ~ 0.03)						
> 20,000 RMB	-0.52	0.26	-1.96	0.053	-0.52 (-1.03 ~ -0.00)						
**Re-admissions within 1 year of discharge**	-0.34	0.14	-2.45	**0.016**	-0.34 (-0.61 ~ -0.07)		-0.16	0.12	-1.39	0.169	-0.16 (-0.39 ~ 0.07)
**Feeding practices**											
Normal feeding					0.00 (Reference)						0.00 (Reference)
Nasogastric/enteric tube feeding	-1.18	0.18	-6.49	**< 0.001**	-1.18 (-1.53 ~ -0.82)		-0.13	0.26	-0.48	0.631	-0.13 (-0.64 ~ 0.38)
Normal feeding mixed nasogastric/enteric tube feeding	-1.24	0.27	-4.58	**< 0.001**	-1.24 (-1.77 ~ -0.71)		-0.53	0.28	-1.89	0.063	-0.53 (-1.07 ~ 0.02)
Normal feeding mixed gastrostomy feeding	-1.76	0.27	-6.52	**< 0.001**	-1.76 (-2.29 ~ -1.23)		-0.61	0.33	-1.85	0.069	-0.61 (-1.25 ~ 0.04)
**Age**	0.08	0.02	3.29	**0.001**	0.08 (0.03 ~ 0.13)		0.06	0.02	3.14	**0.002**	0.06 (0.02 ~ 0.10)
**Barthel Self-Care Rating score**	0.02	0.00	8.95	**< 0.001**	0.02 (0.02 ~ 0.03)		0.02	0.00	4.55	**< 0.001**	0.02 (0.01 ~ 0.03)

## Discussion

This study showed that the mortality rate of children on long-term mechanical ventilation within one year after discharge was 10.38%, slightly lower than the internationally reported rate ranging from 15 to 27% [10–14]. The possible reasons analyzed were the following: the child’s level of self-care was fair, the number of readmissions was low, and the procedure for managing the child’s post-discharge follow-up was appropriate. As early as 2001, Orem’s theory stated that self-care helps to manage health conditions and maintain health [15]. Self-care and self-management are essential to improve outcomes for children with long-term or chronic illnesses [16], which leads to their active participation in home care and improves QoL [17]. The Barthel Self-Care Rating scores of the children in this study were generally at a moderate level of dependence, and the children in the survival group were at a mild to moderate level of dependence, with some self-care in daily life. It can be inferred that children with higher levels of self-care may be better adapted to long-term mechanical ventilation, similar to the findings of Nematollahi M et al. [17]. Nematollahi M et al. also noted that effective self-care is essential in achieving good outcomes and preventing recurrent hospital admissions [17]. Readmission is one of the most critical risk factors for 1-year mortality in patients with sepsis, according to a study by Dashefsky HS et al. [18]. In this study, children who were not readmitted within 90 days of discharge had a 97% lower risk of death compared to those who were readmitted, suggesting that improving self-care of children and decreasing the number of recurrent admissions are strongly associated with good outcomes. This is similar to the findings of Fry CH et al. on the relationship between repeated readmissions due to the same condition and mortality, where frequent readmission events for the exact cause, especially for chronic or age-related conditions, were associated with an increased risk of death within 30 days, six months, and two years after discharge [19].

Additionally, essential factors in reducing mortality within one year after discharge of a child on long-term mechanical ventilation include the procedure for managing the child’s post-discharge follow-up. In the Department of Critical Care Medicine of CHFU, standard procedures have been developed for family continuity of care and follow-up management of children with PMV. Children on long-term mechanical ventilation were confirmed to be included in the follow-up cohort. A file was established before discharge, a trial of the home ventilator was initiated one week before discharge, and parameters were adjusted until the child tolerated them. At least two home caregivers were trained to recognize and handle ventilator alarms, suctioning, daily tracheostomy care, basic life support, and outpatient follow-up procedures. The trainers were PICU respiratory therapists and advanced practice nurses, and the training included one-on-one instruction and bedside scenarios. The duration of the training was approximately 2–4 weeks, and a training manual was issued for review. A contact center was also set up through an online platform to facilitate the exchange of information among caregivers and between caregivers and health workers. In the follow-up management, the child’s caregiver was asked to fill out a follow-up card to report on all aspects of mechanical ventilation in the home. The children were followed up in the outpatient clinic at two weeks, four weeks, three months, and six months after discharge from the hospital and then every six months after their condition stabilized. A multidisciplinary approach was adopted for the follow-up visits, led by critical care respiratory physicians, respiratory therapists, and advanced practice nurses, with the participation of the nutrition department, the rehabilitation department, and the ventilator engineers, who were able to obtain detailed information about the status of the children’s mechanical ventilation at home using follow-up cards. At the same time, the advanced practice nurses will make telephone follow-up visits twice a month to understand the children’s home care situation and provide timely guidance. This standardized follow-up procedure and the multidisciplinary diagnostic and treatment team provide for the transition of children on long-term mechanical ventilation to home mechanical ventilation, ensuring the continuity and effectiveness of the treatment strategy for children on long-term mechanical ventilation.

Quality of life is a vital indicator of treatment effectiveness and one of the most critical outcomes for assessing health status in any chronic disease. It is increasingly used and recommended in clinical treatment and home care [20, 21]. Children with PMV who survived one year after discharge in this study were categorized into a tracheotomy group and a home mechanical ventilation group. Tracheotomy was not associated with higher or lower QoL scores in children but was significantly higher in the non-home mechanical ventilation group. Home invasive mechanical ventilation, age, and Barthel Self-Care Rating score were independent influences. Home invasive mechanical ventilation, younger age, and lower Barthel self-care score were associated with lower QoL scores. Among the children who underwent home mechanical ventilation in this study, 31 (81.58%) underwent invasive mechanical ventilation, with an average ventilation duration of 19.94 h/d. The primary mode of ventilation was the pressure-controlled mode, which was similar to the findings of Zhang Zhengzheng et al. that in China, the majority of prolonged mechanically ventilated children were discharged from the hospital and received invasive mechanical ventilation at home (327, 94.5%) [22]. This is in contrast to the results of international studies. A global report on the evolution of HMV spanning 24 years noted that the number and proportion of children using home noninvasive mechanical ventilation (NIV) were significantly higher than that of children using invasive mechanical ventilation (*n* = 6362 vs. 2453; 72% vs. 28%). Especially in recent years, there has been a strong trend towards initiating home noninvasive mechanical ventilation rather than invasive mechanical ventilation (19% IMV vs. 81% NIV in 2020–2023), especially in Europe, Canada, and South America [1]. This can result from the complex and interactive nature of healthcare infrastructure, the healthcare insurance system, healthcare practitioner choices, and patient family support [23]. A study by Huttmann SE et al. comparing the QoL of patients receiving home noninvasive and invasive mechanical ventilation found that they both had fair QoL but that the QoL of patients receiving home invasive mechanical ventilation was significantly lower and influenced by primary disease. Patients with lower respiratory disease had poorer quality of life and lower long-term survival than those with neuromuscular disease [24]. However, primary disease was not a factor influencing the QoL scores in this study, and we analyzed that it might be related to the small overall sample size and the uneven distribution of various types of primary diseases. In contrast, the study by Valko L et al. showed that QoL does not depend on the mode of home mechanical ventilation and that QoL can be improved even in patients undergoing invasive home mechanical ventilation. This can be attributed to customizing home mechanical ventilation therapy for patients. They develop complex ventilation and rehabilitation programs for patients and follow up patients with home invasive mechanical ventilation monthly [25]. This provides new ideas for continuous improvement of the follow-up management program. Whether we can use home noninvasive mechanical ventilation more often, depending on the type of primary disease and the status of the patient’s disease course, must be further validated. In addition, this study noted that the younger the age of the child on home mechanical ventilation, the lower the QoL score, which is contrary to the findings of Mattson J [26], González R [4] et al. The analysis of the possible reason for this is that the QoL scale for children aged 2 months-5 years in this study used the parental proxy report version. It has been suggested that parent-reported children’s QoL scores are generally lower than children’s self-reports, possibly due to parental experiences while caring for their children at home influencing the decline in parental quality of life, which in turn influences the results of parental proxy reports [4, 27]. As a next step, we can conduct a comparative study to investigate the quality of life of parents of mechanically ventilated children at home in China and its impact on the outcome of the children.

## Conclusion

The long-term survival rate of children on prolonged mechanical ventilation in Shanghai, China, who were transitioned to home mechanical ventilation after discharge was higher than that of the international average, but unlike international reports, most of the children were discharged to home invasive mechanical ventilation, and it resulted in a decrease in QoL. In the future, there is a need to continue standardizing the post-discharge management procedures and explore how to better transition to home non-invasive mechanical ventilation. There is also a need to describe the QoL of parents of children on home mechanical ventilation and its impact on child outcomes or trends in child QoL scores at different times after discharge.

## Data Availability

Data are available from the authors upon reasonable request and with permission of the Children’s Hospital of Fudan University.

## Abbreviations

HRQoL: Health-related quality of life
PMV: Prolonged mechanical ventilation
HMV: Home mechanical ventilation
PICU: Pediatric Intensive Care Unit
CHFU: Children’s Hospital of Fudan University
QoL: Quality of life
TACQOL: TNO-AZL Children’s Health-Related Quality of Life
TAPQOL: TNO-AZL Preschool Children Quality of Life
NIV: Noninvasive mechanical ventilation
IMV: Invasive mechanical ventilation
